# 7-Bromo-2-methyl-1-(phenyl­sulfon­yl)naphtho[2,1-*b*]furan

**DOI:** 10.1107/S160053680801177X

**Published:** 2008-04-30

**Authors:** Hong Dae Choi, Pil Ja Seo, Byeng Wha Son, Uk Lee

**Affiliations:** a Department of Chemistry, Dongeui University, San 24 Kaya-dong, Busanjin-gu, Busan 614-714, Republic of Korea; b Department of Chemistry, Pukyong National University, 599-1 Daeyeon 3-dong, Nam-gu, Busan 608-737, Republic of Korea

## Abstract

The title compound, C_19_H_13_BrO_3_S, was prepared by the oxidation of 7-bromo-2-methyl-1-(phenyl­sulfan­yl)naph­tho[2,1-*b*]furan with 3-chloro­peroxy­benzoic acid. The phenyl ring makes a dihedral angle of 80.4 (2)° with the plane of the naphthofuran fragment. The crystal structure is stabilized by aromatic π–π stacking inter­actions between the brominated benzene ring and the central benzene ring of the naphthofuran system of neighbouring mol­ecules; the centroid–centroid distances within the stack are 3.889 (3) and 3.981 (3) Å. In addition, the stacked mol­ecules exhibit C—H⋯π, inter- and intra­molecular C—H⋯O inter­actions.

## Related literature

For the crystal structures of similar 7-bromo­naphtho[2,1-*b*]furan compounds, see: Choi *et al.* (2006 ▶, 2007 ▶).
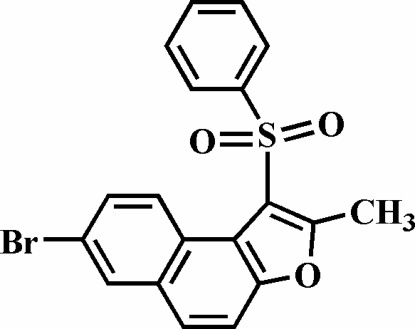


## Experimental

### 

#### Crystal data

C_19_H_13_BrO_3_S
*M* *_r_* = 401.26
Triclinic, 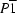

*a* = 7.8583 (7) Å
*b* = 8.0025 (7) Å
*c* = 13.278 (1) Å
α = 107.429 (1)°
β = 93.678 (1)°
γ = 90.417 (2)°
*V* = 794.72 (12) Å^3^
*Z* = 2
Mo *K*α radiation
μ = 2.73 mm^−1^
*T* = 173 (2) K
0.40 × 0.30 × 0.20 mm

#### Data collection

Bruker SMART CCD diffractometer
Absorption correction: multi-scan (*SADABS*; Sheldrick, 2000 ▶) *T*_min_ = 0.381, *T*_max_ = 0.571
6371 measured reflections
3084 independent reflections
2874 reflections with *I* > 2σ(*I*)
*R*_int_ = 0.016

#### Refinement

*R*[*F* ^2^ > 2σ(*F* ^2^)] = 0.023
*wR*(*F* ^2^) = 0.078
*S* = 1.17
3084 reflections
218 parameters
H-atom parameters constrained
Δρ_max_ = 0.44 e Å^−3^
Δρ_min_ = −0.35 e Å^−3^

### 

Data collection: *SMART* (Bruker, 2001 ▶); cell refinement: *SAINT* (Bruker, 2001 ▶); data reduction: *SAINT*; program(s) used to solve structure: *SHELXS97* (Sheldrick, 2008 ▶); program(s) used to refine structure: *SHELXL97* (Sheldrick, 2008 ▶); molecular graphics: *ORTEP-3* (Farrugia, 1997 ▶) and *DIAMOND* (Brandenburg, 1998 ▶); software used to prepare material for publication: *SHELXL97*.

## Supplementary Material

Crystal structure: contains datablocks global, I. DOI: 10.1107/S160053680801177X/jh2063sup1.cif

Structure factors: contains datablocks I. DOI: 10.1107/S160053680801177X/jh2063Isup2.hkl

Additional supplementary materials:  crystallographic information; 3D view; checkCIF report

## Figures and Tables

**Table 1:** Hydrogen-bond geometry (Å, °)

*D*—H⋯*A*	*D*—H	H⋯*A*	*D*⋯*A*	*D*—H⋯*A*
C7—H7⋯*Cg*1^i^	0.95	2.85	3.764 (3)	161
C4—H4⋯O2	0.95	2.44	3.226 (3)	140
C16—H16⋯O3^ii^	0.95	2.56	3.257 (3)	130
C19—H19*A*⋯O3	0.98	2.28	2.926 (3)	122

Symmetry codes: (i) 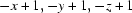; (ii) 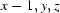. *Cg*1 is the centroid of the C13–C18 ring.

**Fig. 1.**
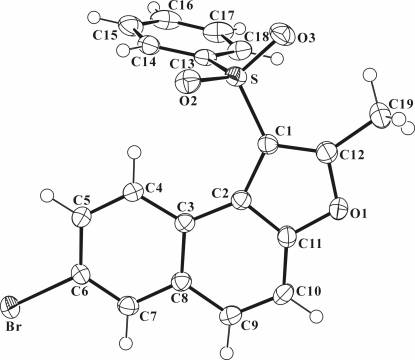
The molecular structure of the title compound, showing displacement ellipsoids drawn at the 50% probability level.

**Fig. 2.**
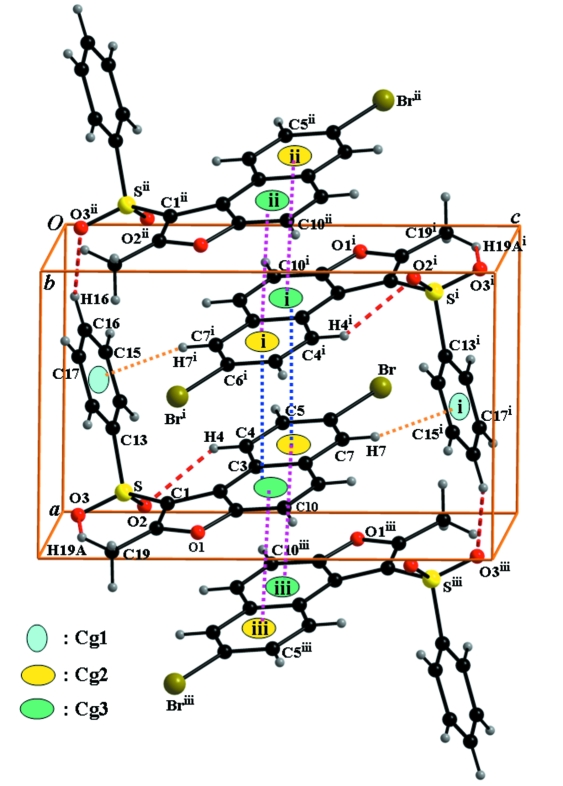
π—π, C—H···π and C—H···O interactions (dotted lines) in the title compound. Cg denotes the ring centroids. [Symmetry code: (i) -x + 1, -y + 1, -z + 1; (ii) x - 1, y, z; (iii)-x + 2,-y + 1,-z + 1.]

## supplementary crystallographic information

### Comment

This work is related to our communications on the synthesis and structures of 7-bromonaphtho[2,1-*b*]furan analogues, *viz*. 7-bromo-1-methylsulfanyl-2-phenylnaphtho[2,1-*b*]furan (Choi *et al.*, 2006) and 7-bromo-2-methyl-1-(phenylsulfanyl)naphtho[2,1-*b*]furan (Choi *et al.*, 2007). Herein we report the molecular and crystal structure of the title compound, 7-bromo-2-methyl-1-(phenylsulfonyl)naphtho[2,1-*b*]furan (Fig. 1).

The naphthofuran unit is essentially planar, with a mean deviation of 0.012 Å from the least-squares plane defined by the thirteen constituent atoms. The phenyl ring (C13—C18) makes a dihedral angle of 80.4 (2)° with the plane of the naphthofuran fragment. The crystal packing (Fig. 2) is stabilized by two π—π stacking interactions within each stack of molecules; one between the brominated benzene ring (*Cg*2) and the central benzene ring (*Cg*3^i^) of an adjacent naphthofuran unit {distance; 3.981 (3) Å}, and a second between the brominated benzene ring (*Cg*2) and the central benzene ring (*Cg*3^iii^) of an adjacent naphthofuran unit {distance; 3.889 (3) Å} (*Cg*2 and *Cg*3 are the centroids of the C3—C8 benzene ring and the C2/C3/C8/C9/C10/C11 benzene ring, respectively, symmetry code as in Fig. 2). The molecular packing is further stabilized by C—H···π interactions between a brominated benzene H atom and the phenyl ring of the phenylsulfonyl substituent, with a C7—H7···*Cg*1^i^ separation of 2.85 Å (Fig. 2 and Table 1; *Cg*1 is the centroid of the C13–18 phenyl ring; symmetry code as in Fig. 2). Additionally, inter- and intramolecular C—H···O interactions in the structure were observed (Fig. 2 and Table 1; symmetry code as in Fig. 2).

### Experimental

3-Chloroperoxybenzoic acid (77%, 320 mg, 1.43 mmol) was added in small portions to a stirred solution of 7-bromo-2-methyl-1-(phenylsulfanyl)naphtho[2,1-*b*]furan (240 mg, 0.65 mmol) in dichloromethane (20 ml) at 273 K. After being stirred at room temperature for 4 h, the mixture was washed with saturated sodium bicarbonate solution and the organic layer was separated, dried over magnesium sulfate, filtered and concentrated in vacuum. The residue was purified by column chromatography (hexane-ethyl acetate, 2: 1 *v*/*v*) to afford the title compound as a colorless solid [yield 81%, m.p. 483–484 K; *R*_f_ = 0.68 (hexane-ethyl acetate, 2:1 *v*/*v*)]. Single crystals suitable for X-ray diffraction were prepared by slow evaporation of a solution of the title compound in benzene at room temperature. Spectroscopic analysis: ^1^H NMR (CDCl_3_, 400 MHz) δ 2.99 (s, 3H), 7.43–7.55 (m, 3H), 7.59–7.71 (m, 3H), 7.95 (d, J = 8.40 Hz, 2H), 8.03 (s, 1H), 8.89 (s, J = 9.16 Hz, 1H); EI—MS 402 [*M*+2], 400 [*M*^+^].

### Refinement

All H atoms were positioned geometrically and refined using a riding model, with C—H = 0.95 Å for aromatic H atoms and 0.98 Å for methyl H atoms, respectively, and with *U*_iso_(H) = 1.2Ueq(C) for aromatic and *U*_iso_(H) = 1.5Ueq(C) for methyl H atoms.

### Crystal data

**Table:**

C_19_H_13_BrO_3_S	*Z* = 2
*M**_r_* = 401.26	*F*_000_ = 404
Triclinic, *P*1	*D*_x_ = 1.677 Mg m^−^^3^
Hall symbol: -p 1	Melting point = 483–484 K
*a* = 7.8583 (7) Å	Mo *K*α radiation λ = 0.71073 Å
*b* = 8.0025 (7) Å	Cell parameters from 4679 reflections
*c* = 13.278 (1) Å	θ = 2.6–28.2º
α = 107.429 (1)º	µ = 2.73 mm^−^^1^
β = 93.678 (1)º	*T* = 173 (2) K
γ = 90.417 (2)º	Block, colorless
*V* = 794.72 (12) Å^3^	0.40 × 0.30 × 0.20 mm

### Data collection

**Table:**

Bruker SMART CCD diffractometer	3084 independent reflections
Radiation source: fine-focus sealed tube	2874 reflections with *I* > 2σ(*I*)
Monochromator: graphite	*R*_int_ = 0.016
Detector resolution: 10.0 pixels mm^-1^	θ_max_ = 26.0º
*T* = 173(2) K	θ_min_ = 1.6º
φ and ω scans	*h* = −9→9
Absorption correction: multi-scan(SADABS; Sheldrick, 2000)	*k* = −9→9
*T*_min_ = 0.381, *T*_max_ = 0.571	*l* = −16→16
6371 measured reflections	

### Refinement

**Table:**

Refinement on *F*^2^	Secondary atom site location: difference Fourier map
Least-squares matrix: full	Hydrogen site location: inferred from neighbouring sites
*R*[*F*^2^ > 2σ(*F*^2^)] = 0.023	H-atom parameters constrained
*wR*(*F*^2^) = 0.078	*w* = 1/[σ^2^(*F*_o_^2^) + (0.0451*P*)^2^ + 0.2317*P*] where *P* = (*F*_o_^2^ + 2*F*_c_^2^)/3
*S* = 1.17	(Δ/σ)_max_ = 0.001
3084 reflections	Δρ_max_ = 0.44 e Å^−^^3^
218 parameters	Δρ_min_ = −0.35 e Å^−^^3^
Primary atom site location: structure-invariant direct methods	Extinction correction: none

### Special details

**Table:**

Geometry. All e.s.d.'s (except the e.s.d. in the dihedral angle between two l.s. planes) are estimated using the full covariance matrix. The cell e.s.d.'s are taken into account individually in the estimation of e.s.d.'s in distances, angles and torsion angles; correlations between e.s.d.'s in cell parameters are only used when they are defined by crystal symmetry. An approximate (isotropic) treatment of cell e.s.d.'s is used for estimating e.s.d.'s involving l.s. planes.
Refinement. Refinement of *F*^2^ against ALL reflections. The weighted *R*-factor *wR* and goodness of fit *S* are based on *F*^2^, conventional *R*-factors *R* are based on *F*, with *F* set to zero for negative *F*^2^. The threshold expression of *F*^2^ > σ(*F*^2^) is used only for calculating *R*-factors(gt) *etc*. and is not relevant to the choice of reflections for refinement. *R*-factors based on *F*^2^ are statistically about twice as large as those based on *F*, and *R*- factors based on ALL data will be even larger.

### Fractional atomic coordinates and isotropic or equivalent isotropic displacement parameters (Å^2^)

**Table:**

	*x*	*y*	*z*	*U*_iso_*/*U*_eq_	
Br	0.56455 (3)	0.99911 (3)	0.696889 (17)	0.03340 (10)	
S	0.82852 (6)	0.36218 (7)	0.16782 (4)	0.02320 (13)	
O1	0.91789 (19)	0.0602 (2)	0.34760 (12)	0.0280 (3)	
O2	0.9034 (2)	0.5362 (2)	0.20422 (12)	0.0303 (3)	
O3	0.8886 (2)	0.2407 (2)	0.07411 (12)	0.0330 (4)	
C1	0.8504 (3)	0.2648 (3)	0.26930 (16)	0.0228 (4)	
C2	0.8191 (2)	0.3362 (3)	0.38127 (16)	0.0216 (4)	
C3	0.7591 (2)	0.4950 (3)	0.45075 (16)	0.0212 (4)	
C4	0.7119 (3)	0.6446 (3)	0.42140 (17)	0.0248 (4)	
H4	0.7201	0.6435	0.3501	0.030*	
C5	0.6545 (3)	0.7918 (3)	0.49309 (17)	0.0265 (4)	
H5	0.6230	0.8911	0.4717	0.032*	
C6	0.6432 (3)	0.7931 (3)	0.59824 (17)	0.0245 (4)	
C7	0.6885 (3)	0.6539 (3)	0.63190 (17)	0.0256 (4)	
H7	0.6804	0.6593	0.7039	0.031*	
C8	0.7476 (3)	0.5013 (3)	0.55938 (16)	0.0229 (4)	
C9	0.7963 (3)	0.3570 (3)	0.59591 (17)	0.0265 (4)	
H9	0.7877	0.3654	0.6683	0.032*	
C10	0.8549 (3)	0.2078 (3)	0.52932 (17)	0.0269 (5)	
H10	0.8883	0.1115	0.5533	0.032*	
C11	0.8634 (3)	0.2034 (3)	0.42350 (17)	0.0241 (4)	
C12	0.9097 (3)	0.0997 (3)	0.25436 (18)	0.0265 (4)	
C13	0.6075 (3)	0.3813 (3)	0.14109 (15)	0.0238 (4)	
C14	0.5417 (3)	0.5444 (3)	0.14748 (17)	0.0294 (5)	
H14	0.6117	0.6475	0.1741	0.035*	
C15	0.3714 (3)	0.5544 (4)	0.11432 (19)	0.0372 (6)	
H15	0.3246	0.6649	0.1178	0.045*	
C16	0.2704 (3)	0.4035 (4)	0.07638 (19)	0.0404 (6)	
H16	0.1546	0.4108	0.0531	0.049*	
C17	0.3368 (3)	0.2418 (4)	0.0720 (2)	0.0395 (6)	
H17	0.2657	0.1392	0.0473	0.047*	
C18	0.5066 (3)	0.2292 (3)	0.10354 (17)	0.0310 (5)	
H18	0.5533	0.1183	0.0995	0.037*	
C19	0.9685 (3)	−0.0435 (3)	0.1644 (2)	0.0379 (6)	
H19A	0.9215	−0.0285	0.0975	0.057*	
H19B	0.9292	−0.1568	0.1697	0.057*	
H19C	1.0933	−0.0395	0.1665	0.057*	

### Atomic displacement parameters (Å^2^)

**Table:**

	*U*^11^	*U*^22^	*U*^33^	*U*^12^	*U*^13^	*U*^23^
Br	0.04740 (16)	0.02425 (14)	0.02669 (14)	0.00861 (10)	0.00501 (10)	0.00413 (9)
S	0.0219 (2)	0.0270 (3)	0.0203 (2)	0.00089 (19)	0.00223 (19)	0.0063 (2)
O1	0.0319 (8)	0.0226 (8)	0.0282 (8)	0.0070 (6)	−0.0002 (6)	0.0061 (6)
O2	0.0302 (8)	0.0308 (8)	0.0310 (8)	−0.0054 (6)	0.0006 (6)	0.0116 (7)
O3	0.0325 (8)	0.0402 (10)	0.0247 (8)	0.0068 (7)	0.0088 (6)	0.0058 (7)
C1	0.0225 (10)	0.0231 (10)	0.0213 (10)	0.0004 (8)	−0.0009 (8)	0.0052 (8)
C2	0.0205 (9)	0.0222 (10)	0.0218 (10)	−0.0009 (8)	−0.0013 (8)	0.0065 (8)
C3	0.0191 (9)	0.0224 (10)	0.0218 (10)	−0.0016 (7)	−0.0017 (7)	0.0067 (8)
C4	0.0300 (11)	0.0240 (10)	0.0215 (10)	0.0013 (8)	0.0002 (8)	0.0086 (8)
C5	0.0331 (11)	0.0215 (10)	0.0254 (11)	0.0017 (8)	−0.0008 (9)	0.0083 (8)
C6	0.0278 (10)	0.0207 (10)	0.0227 (10)	0.0015 (8)	0.0013 (8)	0.0031 (8)
C7	0.0297 (11)	0.0265 (11)	0.0208 (10)	−0.0003 (9)	0.0024 (8)	0.0070 (8)
C8	0.0231 (10)	0.0233 (10)	0.0225 (10)	−0.0010 (8)	−0.0007 (8)	0.0075 (8)
C9	0.0291 (11)	0.0290 (11)	0.0239 (10)	0.0002 (9)	0.0005 (8)	0.0119 (9)
C10	0.0285 (11)	0.0255 (11)	0.0298 (11)	0.0030 (8)	−0.0018 (9)	0.0140 (9)
C11	0.0231 (10)	0.0202 (10)	0.0269 (11)	0.0016 (8)	−0.0017 (8)	0.0047 (8)
C12	0.0239 (10)	0.0276 (11)	0.0262 (10)	0.0025 (8)	−0.0014 (8)	0.0059 (9)
C13	0.0236 (10)	0.0322 (12)	0.0162 (9)	0.0012 (8)	0.0026 (8)	0.0080 (8)
C14	0.0333 (11)	0.0326 (12)	0.0209 (10)	0.0051 (9)	0.0016 (9)	0.0060 (9)
C15	0.0355 (12)	0.0495 (15)	0.0257 (11)	0.0166 (11)	0.0050 (9)	0.0091 (11)
C16	0.0230 (11)	0.0736 (19)	0.0263 (12)	0.0053 (11)	0.0022 (9)	0.0172 (12)
C17	0.0310 (12)	0.0577 (17)	0.0314 (12)	−0.0138 (11)	−0.0041 (10)	0.0174 (12)
C18	0.0332 (12)	0.0355 (13)	0.0261 (11)	−0.0044 (10)	−0.0010 (9)	0.0128 (10)
C19	0.0454 (14)	0.0306 (13)	0.0327 (13)	0.0122 (10)	0.0012 (11)	0.0022 (10)

### Geometric parameters (Å, °)

**Table:**

Br—C6	1.908 (2)	C8—C9	1.425 (3)
S—O2	1.4378 (16)	C9—C10	1.359 (3)
S—O3	1.4400 (16)	C9—H9	0.9500
S—C1	1.748 (2)	C10—C11	1.401 (3)
S—C13	1.767 (2)	C10—H10	0.9500
O1—C12	1.364 (3)	C12—C19	1.487 (3)
O1—C11	1.372 (3)	C13—C14	1.388 (3)
C1—C12	1.366 (3)	C13—C18	1.390 (3)
C1—C2	1.462 (3)	C14—C15	1.392 (3)
C2—C11	1.379 (3)	C14—H14	0.9500
C2—C3	1.432 (3)	C15—C16	1.383 (4)
C3—C4	1.410 (3)	C15—H15	0.9500
C3—C8	1.437 (3)	C16—C17	1.385 (4)
C4—C5	1.375 (3)	C16—H16	0.9500
C4—H4	0.9500	C17—C18	1.386 (3)
C5—C6	1.402 (3)	C17—H17	0.9500
C5—H5	0.9500	C18—H18	0.9500
C6—C7	1.360 (3)	C19—H19A	0.9800
C7—C8	1.413 (3)	C19—H19B	0.9800
C7—H7	0.9500	C19—H19C	0.9800
			
O2—S—O3	118.45 (10)	C9—C10—C11	116.5 (2)
O2—S—C1	109.45 (10)	C9—C10—H10	121.8
O3—S—C1	107.55 (10)	C11—C10—H10	121.8
O2—S—C13	107.64 (10)	O1—C11—C2	111.29 (18)
O3—S—C13	106.29 (10)	O1—C11—C10	122.57 (19)
C1—S—C13	106.88 (10)	C2—C11—C10	126.1 (2)
C12—O1—C11	107.28 (17)	O1—C12—C1	110.04 (19)
C12—C1—C2	107.30 (19)	O1—C12—C19	113.40 (19)
C12—C1—S	122.87 (17)	C1—C12—C19	136.5 (2)
C2—C1—S	129.79 (16)	C14—C13—C18	121.4 (2)
C11—C2—C3	118.02 (18)	C14—C13—S	119.76 (17)
C11—C2—C1	104.08 (18)	C18—C13—S	118.51 (17)
C3—C2—C1	137.90 (19)	C13—C14—C15	119.0 (2)
C4—C3—C2	125.47 (19)	C13—C14—H14	120.5
C4—C3—C8	117.87 (19)	C15—C14—H14	120.5
C2—C3—C8	116.67 (19)	C16—C15—C14	120.0 (2)
C5—C4—C3	121.8 (2)	C16—C15—H15	120.0
C5—C4—H4	119.1	C14—C15—H15	120.0
C3—C4—H4	119.1	C15—C16—C17	120.5 (2)
C4—C5—C6	118.9 (2)	C15—C16—H16	119.7
C4—C5—H5	120.5	C17—C16—H16	119.7
C6—C5—H5	120.5	C16—C17—C18	120.3 (2)
C7—C6—C5	122.2 (2)	C16—C17—H17	119.9
C7—C6—Br	119.45 (16)	C18—C17—H17	119.9
C5—C6—Br	118.36 (16)	C17—C18—C13	118.9 (2)
C6—C7—C8	119.7 (2)	C17—C18—H18	120.6
C6—C7—H7	120.1	C13—C18—H18	120.6
C8—C7—H7	120.1	C12—C19—H19A	109.5
C7—C8—C9	119.17 (19)	C12—C19—H19B	109.5
C7—C8—C3	119.49 (19)	H19A—C19—H19B	109.5
C9—C8—C3	121.34 (19)	C12—C19—H19C	109.5
C10—C9—C8	121.4 (2)	H19A—C19—H19C	109.5
C10—C9—H9	119.3	H19B—C19—H19C	109.5
C8—C9—H9	119.3		
			
O2—S—C1—C12	−131.89 (18)	C8—C9—C10—C11	−0.4 (3)
O3—S—C1—C12	−2.0 (2)	C12—O1—C11—C2	0.2 (2)
C13—S—C1—C12	111.81 (19)	C12—O1—C11—C10	179.8 (2)
O2—S—C1—C2	45.6 (2)	C3—C2—C11—O1	179.89 (17)
O3—S—C1—C2	175.49 (18)	C1—C2—C11—O1	0.0 (2)
C13—S—C1—C2	−70.7 (2)	C3—C2—C11—C10	0.3 (3)
C12—C1—C2—C11	−0.1 (2)	C1—C2—C11—C10	−179.6 (2)
S—C1—C2—C11	−177.90 (16)	C9—C10—C11—O1	−179.10 (19)
C12—C1—C2—C3	180.0 (2)	C9—C10—C11—C2	0.4 (3)
S—C1—C2—C3	2.2 (4)	C11—O1—C12—C1	−0.3 (2)
C11—C2—C3—C4	178.60 (19)	C11—O1—C12—C19	178.94 (19)
C1—C2—C3—C4	−1.5 (4)	C2—C1—C12—O1	0.2 (2)
C11—C2—C3—C8	−1.0 (3)	S—C1—C12—O1	178.21 (14)
C1—C2—C3—C8	178.9 (2)	C2—C1—C12—C19	−178.7 (3)
C2—C3—C4—C5	179.6 (2)	S—C1—C12—C19	−0.7 (4)
C8—C3—C4—C5	−0.8 (3)	O2—S—C13—C14	5.9 (2)
C3—C4—C5—C6	0.2 (3)	O3—S—C13—C14	−121.94 (17)
C4—C5—C6—C7	0.5 (3)	C1—S—C13—C14	123.41 (17)
C4—C5—C6—Br	179.61 (16)	O2—S—C13—C18	179.40 (16)
C5—C6—C7—C8	−0.6 (3)	O3—S—C13—C18	51.54 (19)
Br—C6—C7—C8	−179.70 (15)	C1—S—C13—C18	−63.10 (19)
C6—C7—C8—C9	179.35 (19)	C18—C13—C14—C15	−0.7 (3)
C6—C7—C8—C3	0.0 (3)	S—C13—C14—C15	172.57 (17)
C4—C3—C8—C7	0.7 (3)	C13—C14—C15—C16	0.4 (3)
C2—C3—C8—C7	−179.67 (18)	C14—C15—C16—C17	0.7 (4)
C4—C3—C8—C9	−178.63 (19)	C15—C16—C17—C18	−1.4 (4)
C2—C3—C8—C9	1.0 (3)	C16—C17—C18—C13	1.1 (4)
C7—C8—C9—C10	−179.6 (2)	C14—C13—C18—C17	0.0 (3)
C3—C8—C9—C10	−0.3 (3)	S—C13—C18—C17	−173.39 (17)

### Hydrogen-bond geometry (Å, °)

**Table:**

*D*—H···*A*	*D*—H	H···*A*	*D*···*A*	*D*—H···*A*
C7—H7···Cg1^i^	0.95	2.85	3.764 (3)	161
C4—H4···O2	0.95	2.44	3.226 (3)	140
C16—H16···O3^ii^	0.95	2.56	3.257 (3)	130
C19—H19A···O3	0.98	2.28	2.926 (3)	122

Symmetry codes: (i) −*x*+1, −*y*+1, −*z*+1; (ii) *x*−1, *y*, *z*.

## References

[bb1] Brandenburg, K. (1998). *DIAMOND* Crystal Impact GbR, Bonn, Germany.

[bb2] Bruker (2001). *SAINT* and *SMART* Bruker AXS Inc., Madison, Wisconsin, USA.

[bb3] Choi, H. D., Seo, P. J., Son, B. W. & Lee, U. (2006). *Acta Cryst.* E**62**, o5876–o5877.

[bb4] Choi, H. D., Seo, P. J., Son, B. W. & Lee, U. (2007). *Acta Cryst.* E**63**, o4102.

[bb5] Farrugia, L. J. (1997). *J. Appl. Cryst.* **30**, 565.

[bb6] Sheldrick, G. M. (2000). *SADABS* University of Göttingen, Germany.

[bb7] Sheldrick, G. M. (2008). *Acta Cryst.* A**64**, 112–122. 10.1107/S0108767307043930 18156677

